# Reduced peak oxygen uptake and implications for cardiovascular health and quality of life in patients with schizophrenia

**DOI:** 10.1186/1471-244X-11-188

**Published:** 2011-12-05

**Authors:** Jørn Heggelund, Jan Hoff, Jan Helgerud, Geir E Nilsberg, Gunnar Morken

**Affiliations:** 1 Norwegian University of Science and Technology, Faculty of Medicine, Department of Neuroscience, Trondheim, Norway; 2 St. Olavs University Hospital, Division of Psychiatry, Department of Research and Development (AFFU), Trondheim, Norway; 3 St. Olavs University Hospital, Division of Psychiatry, Department of Østmarka, Trondheim, Norway; 4 Norwegian University of Science and Technology, Faculty of Medicine, Department of Circulation and Medical Imaging, Trondheim, Norway; 5 St.Olavs University Hospital, Department of Physical Medicine and Rehabilitation, Trondheim, Norway; 6 Hokksund Medical Rehabilitation Centre, Hokksund, Norway; 7 Telemark University College, Department of Sports and Outdoor Life Studies, Bø, Norway

## Abstract

**Background:**

Peak oxygen uptake (VO_2peak_) is a strong predictor of cardiovascular disease (CVD) and all-cause mortality, but is inadequately described in patients with schizophrenia. The aim of this study was to evaluate treadmill VO_2peak_, CVD risk factors and quality of life (QOL) in patients with schizophrenia (ICD-10, F20-29).

**Methods:**

33 patients, 22 men (33.7 ± 10.4 years) and 11 women (35.9 ± 11.5 years), were included. Patients VO_2peak _were compared with normative VO_2peak _in healthy individuals from the Nord-Trøndelag Health Study (HUNT). Risk factors were compared above and below the VO_2peak _thresholds; 44.2 and 35.1 ml·kg^-1^·min^-1 ^in men and women, respectively.

**Results:**

VO_2peak _was 37.1 ± 9.2 ml·kg^-1^·min^-1 ^in men with schizophrenia; 74 ± 19% of normative healthy men (p < 0.001). VO_2peak _was 35.6 ± 10.7 ml·kg^-1^·min^-1 ^in women with schizophrenia; 89 ± 25% of normative healthy women (n.s.). Based on odds ratio patients were 28.3 (95% CI = 1.6-505.6) times more likely to have one or more CVD risk factors if they were below the VO_2peak _thresholds. VO_2peak _correlated with the SF-36 physical functioning (r = 0.58), general health (r = 0.53), vitality (r = 0.47), social function (r = 0.41) and physical component score (r = 0.51).

**Conclusion:**

Men with schizophrenia have lower VO_2peak _than the general population. Patients with the lowest VO_2peak _have higher odds of having one or more risk factors for cardiovascular disease. VO_2peak _should be regarded as least as important as the conventional risk factors for CVD and evaluation of VO_2peak _should be incorporated in clinical practice.

## Background

Patients suffering from schizophrenia have a mortality risk that is two to three times that of the general population and the leading cause of death is cardiovascular disease (CVD) [1,2]. Although, multifactor causes have been identified, reduced cardiorespiratory fitness has probably been overlooked as a risk factor for CVD in patients with schizophrenia [3].

Cardiorespiratory fitness, measured as peak oxygen uptake (VO_2peak_) is a strong predictor of CVD and all-cause mortality [4,5]. Improvements in VO_2peak _have indicated reduced risk of CVD, coronary heart disease and all cause mortality [5]. VO_2peak _is often a stronger predictor of mortality than conventional risk factors for CVD [6]. McAuley and Blair [7] recently pointed out reduced cardiorespiratory fitness as a greater health threat than obesity and suggested that more emphasis should be put on increasing VO_2peak_. This might be especially important considering that higher levels of VO_2peak _seems to attenuate or eliminate the increased health risk associated with obesity [8]. Findings from the epidemiological Nord-Trøndelag Health Study (the HUNT Study) demonstrate that physical active people with a clustering of cardiovascular risk factors appears to have comparable risk of premature death as inactive individuals without risk factors [9]. In the same cohort men with VO_2peak _below 44.2 ml·kg^-1^·min^-1 ^were eight times more likely to have a cluster of CVD risk factors, compared to men above 50.5 ml·kg^-1^·min^-1 ^[10].

Results from the Aerobics Center Longitudional Study further suggest that people with low VO_2peak _is characterized by depressive symptoms and low emotional well being [11]. High levels of VO_2peak _are associated with high levels of quality of life (QOL) [12]. Body mass index (BMI) are found inversely related to QOL in patients with schizophrenia [3] but the relation between VO_2peak _and perceived QOL are not evaluated.

Objective measures of VO_2peak _have rarely been presented in patients with schizophrenia. The classical study by Carlson et al. [13] were the first to describe oxygen uptake in patients with schizophrenia, but many of their patients did not reach values close to maximal oxygen uptake. Our research group revealed significant changes in VO_2peak _after eight weeks of high aerobic intensity training in patients with schizophrenia [14]. Recently, Strassnig et al. [3] published measures of oxygen uptake in 117 patients with schizophrenia that were exceedingly low (4.4 metabolic equivalents ≈ 15.4 ml·kg^-1^·min^-1^). This VO_2peak _value are much lower than the VO_2 _required for walking in patients with schizophrenia [14], and at a level that may indicate a need for heart transplant in heart failure patients [15].

The primary aim of this study was to evaluate objectively measured VO_2peak _during walking or running in men and women with schizophrenia compared to VO_2peak _in healthy individuals from the Nord-Trøndelag Health Study (HUNT). We hypothesized that patients with schizophrenia had reduced VO_2peak _compared to normative healthy individuals. The secondary aim was to evaluate relationships between VO_2peak_, risk factors for cardiovascular disease, and quality of life.

## Methods

### Subjects

We included 33 patients, 11 women and 22 men, with ICD-10 schizophrenia, schizotypal or delusional disorders (F20 to F29) in the study. Patients were in- and out-patients at a University hospital and had agreed to take part in exercise interventions studies. All patients were under antipsychotic medical treatment. 24 patients were smokers. Exclusion criteria were known coronary artery disease, known chronic obstructive pulmonary disease, and not being able to perform physical treadmill testing and exercise. Patients were examined by a physician at inclusion to the study and the exclusion criterions were confirmed by medical records.

### Assessments

An individualized protocol was applied to measure VO_2peak _and peak heart rate (HR_peak_), using the Cortex Metamax II portable metabolic test system (Cortex Biophysik GmbH, Leipzig, Germany) and the Polar S610i heart rate monitor (Polar Electro, Finland), respectively. The protocol has previously been described in patients with schizophrenia as well as in healthy individuals [14,16].

The patients were carefully familiarized with the test procedures and the treadmill when entering the laboratory. Warm-up was ten minute walking or running on the treadmill at an intensity corresponding to 60-70% HR_peak_. The test started from warm-up speed (with minimum 5% inclination) after which the speed or the inclination was increased every minute (0.5-1 km·h^-1 ^and 1-2%, respectively) to a level that brought the patient to exhaustion. The highest oxygen uptake and heart rate (HR) recorded during the last minute of the test were determined as VO_2peak _and HR_peak_, respectively. VO_2peak _where also presented as ml·kg^-0.75^·min^-1 ^to normalise for the differences in bodyweight between the patients [17].

We compared the patients VO_2peak _with age and sex specific strata from the Nord-Trøndelag Health Study (the HUNT Study) [10]. The HUNT study is an epidemiological study of the general population in the neighbouring county to the university hospital. The HUNT Fitness study tested VO_2peak _in 4 631 healthy individuals (20 to 90 years) using mixing chamber gas-analyzer ergospirometry (Cortex MetaMax II, Cortex, Leipzig, Germany) and an individualised protocol that has close resemblance to the protocol used in the present study. 14.1% of the participants reported to be inactive, defined as no activity or exercising less than once per week. For each patient with schizophrenia, we estimated a normative VO_2peak_, namely the mean value defined in the HUNT Fitness study strata for the corresponding sex and age. We titled the VO_2peak _estimated from sex and age strata independent of physical activity level, as HUNT general. The VO_2peak _from age and sex strata for healthy inactive men and women were titled HUNT inactive. The percent of HUNT general and HUNT inactive VO_2peak _was calculated as: (achieved VO_2peak _÷ age predicted VO_2peak_) · 100.

In the HUNT Fitness study men and women below 44.2 ml·kg^-1^·min^-1 ^and 35.1 ml·kg^-1^·min^-1^, respectively, were associated with higher cardiovascular risk factor profile [10]. The same VO_2peak _values were used as threshold values when evaluating conventional CVD risk factors.

Morning fasting blood levels were taken. Serum glucose was analysed using Reflotron Plus system (Roche Diagnostics, Mannheim, Germany). HDL (high-density-lipoprotein) cholesterol, total cholesterol and triglyceride concentrations in serum were measured using a Modular P chemistry analyzer (Roche Diagnostics, Mannheim, Germany). LDL cholesterol was calculated using the Friedewald equation [18]. BP (blood pressure) was measured using a Maxi-Stabil 3 (Welch Allyn, Jungingen, Germany). Patients were sitting and had rested for at least 5 minutes. Risk factors were classified as follows: hypertension, diastolic pressure ≥ 90 mmHg and/or systolic pressure ≥ 140 mmHg; elevated blood glucose, > 6.0 mmol·L^-1^; elevated total cholesterol, > 6.1 mmol·L^-1 ^in patients < 30 years old, > 6.9 mmol·L^-1 ^in patients 30-49 years old and > 7.8 mmol·L^-1 ^in patients ≥ 50 years old; elevated LDL-cholesterol, 4.3 > mmol·L^-1 ^in patients < 30 years old, 4.7 > mmol·L^-1 ^in patients 30-49 years old and > 5.3 mmol·L^-1 ^in patients ≥ 50 years old; reduced HDL-cholesterol, < 1.0 mmol·L^-1^; elevated triglyceride, > 2.6 mmol·L^-1^; obesity, BMI ≥ 30.0 kg·m^-1 ^[19,20].

The short form (SF-36) was used to assess the physical health and mental health aspects of health related quality of life [21]. SF-36 consists of eight sub scores and can also be divided into a physical component score (PCS) and mental component score (MCS). 0 reflect the poorest health whereas 100 reflect the best health.

The Positive and Negative Syndrome Scale (PANSS) was used to evaluate the severity of symptoms of schizophrenia [22]. PANSS constitutes three scales measuring positive (productive symptoms), negative symptoms (deficit features) and general severity of illness. A total of 30 items are evaluated on a likert scale ranging from 1 (absent) to 7 (extreme) and added up to a total score as well as the three sub scores. In this study we used the positive and negative sub scores (7 items each) as well as the total score (30 items).

### Analyses

We used the independent samples T-test to compare differences between men and women, between patients below and above the VO_2peak _thresholds as well as between measured VO_2peak _and HUNT general and HUNT inactive VO_2peak_. We used the Pearson chi-square test to detect whether there was a significant association between patients above/below the VO_2peak _threshold and prevalence of risk factors. We calculated the odds ratio for having one or more risk factors in the patients below threshold. The analysis was adjusted for age and sex. In multiadjusted analysis we also adjusted for the potential cofounding effect of smoking.

We used Pearson r to analyse correlations between VO_2peak _(ml·kg^-0.75^·min^-1^) and each domain of the SF-36. The significance level (α) was set at p *<*0.05 (2-tailed). Data are described as mean and standard deviation (SD), unless otherwise noted. SPSS statistical package, version 18.0 (SPSS Inc.), was applied to analyse results.

The study was approved by the regional committees for medical and health research ethics, middle Norway and conducted according to the Helsinki declaration. Written informed consent was obtained from all the included patients after the procedures were fully explained.

## Results

### Demographics

Age was 33.7 ± 10.4 years and 35.9 ± 11.5 years in men and women, respectively. The total PANSS, total positive PANSS and total negative PANSS score was 65 ± 17, 15 ± 6 and 17 ± 8 in men, and 68 ± 23, 16 ± 6 and 18 ± 8 in women, respectively.

### Peak oxygen uptake

**Figure 1:**
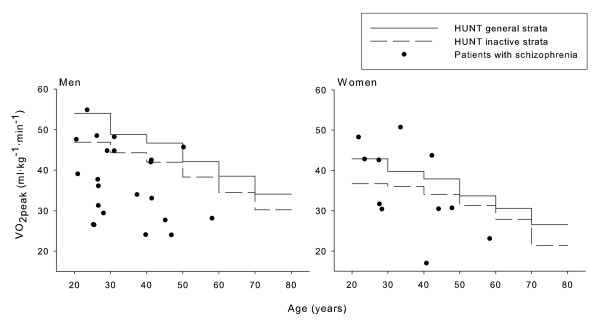
**Peak oxygen uptake in patients with schizophrenia and normative healthy men and women**. Normative strata are adopted from the HUNT fitness study [10]. HUNT general strata are age and sex specific strata regardless of physical activity level.

The VO_2peak _for the men and women with schizophrenia are presented in Table 1. Individual VO_2peak _values are plotted against age as well as normative VO_2peak _strata from the HUNT Fitness study in Figure 1. VO_2peak _in the men with schizophrenia was 84 ± 21% of age predicted HUNT inactive (p < 0.001) and 74 ± 19% of HUNT general (p < 0.001). The VO_2peak _in the women with schizophrenia was not different from HUNT inactive (101 ± 28%) and HUNT general (89 ± 25%; n.s.). Age predicted VO_2peak _was 44.5 ± 2.9 in HUNT inactive men, 50.3 ± 4.1 ml·kg^-1^·min^-1 ^in HUNT general men, 35.2 ± 1.8 in HUNT inactive women and 40.0 ± 3.2 ml·kg^-1^·min^-1 ^in HUNT general women.

**Table 1:** Individual characteristics from the peak oxygen uptake test

Patient	Age	BW	**VO**_**2peak**_	**VO**_**2peak**_	**VO**_**2peak**_	**V**_**E**_	RER	HR
**nr**	**years**	**Kg**	**L·min**^**-1**^	**ml·kg^-1 ^·min**^**-1**^	**ml·kg^-0.75 ^·min**^**-1**^	**L·min**^**-1**^		**Beats ·min**^**-1**^

Men								
1	21	76.5	3.64	47.6	140.7	126.1	1.07	186
2	21	95.3	3.72	39.1	122.0	107.7	1.09	175
3	24	66.6	3.65	54.9	156.6	138.1	1.19	211
4	25	159.5	4.25	26.6	94.7	134.0	1.19	154
5	25	120.9	3.21	26.5	88.0	115.8	1.09	160
6	26	65.0	3.15	48.5	137.6	100.4	1.22	185
7	26	107.9	4.07	37.7	121.6	126.3	1.23	177
8	27	96.7	3.50	36.1	113.5	102.4	1.11	186
9	27	107.2	3.36	31.3	100.9	94.7	1.04	180
10	28	100.6	2.96	29.4	93.2	69.2	1.00	190
11	29	66.7	2.99	44.8	128.1	121.7	1.13	183
12	31	78.0	3.76	48.2	143.3	118.6	1.41	188
13	31	70.1	3.14	44.8	129.6	106.9	1.10	165
14	37	114.0	3.87	34.0	110.9	109.2	1.10	153
15	40	98.7	2.38	24.1	76.0	65.2	1.00	143
16	41	77.4	3.29	42.0	126.1	110.4	1.21	153
17	41	89.5	3.80	42.5	130.6	105.7	1.22	164
18	41	117.1	3.88	33.1	109.0	118.6	1.06	165
19	45	122.3	3.38	27.7	91.9	125.4	1.08	150
20	47	122.7	2.94	24.0	79.7	89.2	1.00	153
21	50	75.7	3.46	45.7	134.8	90.8	1.08	156
22	58	109.6	3.07	28.1	90.6	91.2	1.07	160

Mean ± SD	33.7 ± 10.4	97.2 ± 24.0	3.43 ± 0.44	37.1 ± 9.2	114.5 ± 22.6	107.6 ± 19.0	1.12 ± 0.10	170 ± 17

Women								
1	22	61.7	2.98	48.3	135.4	94.1	1.18	180
2	24	53.3	2.28	42.8	115.6	54.0	0.99	173
3	28	66.4	2.82	42.6	121.2	97.1	1.14	169
4	28	73.5	2.33	31.7	92.8	66.4	1.20	176
5	28	80.3	2.44	30.4	91.0	93.0	1.20	188
6	34	51.6	2.61	50.7	135.6	79.8	1.27	194
7	41	144.5	2.46	17.0	59.0	71.0	1.02	150
8	42	75.5	3.30	43.7	128.8	108.0	1.20	163
9	44	64.9	1.98	30.5	86.6	56.0	1.16	175
10	48	55.5	1.70	30.7	83.6	73.2	1.10	168
11	58	91.8	2.12	23.1	71.5	74.1	1.13	151

Mean ± SD	35.9 ± 11.5	74.5 ± 26.3	2.46 ± 0.46	35.6 ± 10.7	101.9 ± 26.6	78.8 ± 17.4	1.15 ± 0.08	172 ± 14

BW, body weight; VO_2peak_, peak oxygen uptake; HR, heart rate; V_E_, total pulmonary ventilation; RER, respiratory exchange ratio; SD, standard deviation

### Conventional risk factors

Risk factor assessment was lost in one male patient. Risk factors were present in 24 of 32 patients and of these five were above and 19 were below the thresholds. Among the eight patients without risk factors, six were above and two were below the thresholds (χ^2 ^= 7.6, df = 1, p = 0.006). Based on the odds ratio adjusted for age and sex patients were 24.2 (95% CI = 1.5-505.6) times more likely to have one or more risk factors if they were below the VO_2peak _threshold. When we also adjusted for smoking the odds ratio was 28.3 (95% CI = 1.6-505.6). Among the patients below the VO_2peak _thresholds 10 patients had hypertension, 11 elevated glucose, 12 reduced HDL-cholesterol, 11 elevated triglyceride and 14 had obesity. Above the thresholds 2 patients had hypertension, 2 elevated glucose and 1 was obese. There were 8 smokers above the thresholds and 16 below. Differences in mean levels are presented in Table 2.

**Table 2:** Characteristics^a ^in patients below and above the threshold peak oxygen uptake (VO_2peak_)^b^

	Men		
	**VO_2peak _(< 44.2) (n = 15)**	**VO_2peak _(≥ 44.2) (n = 6)**	**Mean difference (95% CI)**

Systolic pressure (mm Hg)	139.4 ± 16.8	125.7 ± 11.0	-13.8 (-29.6 to 2.0)
Diastolic pressure (mm Hg)	87.4 ± 7.0	84.3 ± 5.0	-3.0 (-9.7 to 3.6)
Total cholesterol (mmol·L^-1^)	5.2 ± 0.7	4.5 ± 0.5	-0.7 (-1.3 to -0.0)
HDL cholesterol (mmol·L^-1^)	1.0 ± 0.3	1.6 ± 0.4	0.5 (0.2 to 0.9)
LDL cholesterol (mmol·L^-1^)	3.2 ± 0.6	2.5 ± 0.6	-0.7 (-1.3 to -0.1)
Triglyceride (mmol·L^-1^)	2.1 ± 1.1	0.9 ± 0.2	-1.2 (-1.8 to -0.6)
Glucose (mmol·L^-1^)	6.4 ± 2.0	5.8 ± 0.6	-0.6 (-2.4 to 1.1)
BMI (kg·m^-2^)	33.0 ± 5.5	23.6 ± 2.4	-9.5 (-14.5 to -4.5)

	Women		

	**VO_2peak_ (<35.1) (n=6)**	**VO_2peak_ (≥35.1) (n=5)**	

Systolic pressure (mm Hg)	121.6 ± 7.9	103.8 ± 11.9	-17.8 (-32.6 to -3.0)
Diastolic pressure (mm Hg)	80.8 ± 4.4	68.8 ± 8.8	-12 (-22.1 to -1.9)
Total cholesterol (mmol·L^-1^)	5.0 ± 0.7	4.6 ± 1.0	-0.4 (-1.5 to 0.8)
HDL cholesterol (mmol·L^-1^)	1.3 ± 0.3	1.8 ± 0.3	0.5 (0.1 to 0.9)
LDL cholesterol (mmol·L^-1^)	2.9 ± 0.9	2.5 ± 1.1	-0.4 (-1.8 to 1.0)
Triglyceride (mmol·L^-1^)	1.8 ± 0.7	0.8 ± 0.3	-1.0 (-1.7 to -0.3)
Glucose (mmol·L^-1^)	6.1 ± 1.5	4.8 ± 0.4	-1.3 (-2.9 to 0.2)
BMI (kg·m^-2^)	31.2 ± 10.9	23.6 ± 4.5	-7.5 (-19.5 to 4.4)

	All		

	< threshold (n = 21)	≥ threshold (n = 11)	

Systolic pressure (mm Hg)	134.7 ± 16.8	115.7 ± 15.7	-19.0 (-31.8 to -6.3)
Diastolic pressure (mm Hg)	85.63 ± 6.9	77.3 ± 10.4	-8.4 (-14.9 to -1.9)
Total cholesterol (mmol·L^-1^)	5.1 ± 0.7	4.6 ± 0.7	-0.6 (-1.1 to -0.0)
HDL cholesterol (mmol·L^-1^)	1.1 ± 0.3	1.6 ± 0.3	0.5 (0.0 to 0.1)
LDL cholesterol (mmol·L^-1^)	3.1 ± 0.7	2.5 ± 0.8	-0.6 (-1.2 to -0.1)
Triglyceride (mmol·L^-1^)	2.0 ± 1.0	0.9 ± 0.3	-1.1 (-1.6 to -0.7)
Glucose (mmol·L^-1^)	6.3 ± 1.8	5.3 ± 0.7	-1.0 (-2.2 to 0.2)
BMI (kg·m^-2^)	32.4 ± 7.5	23.6 ± 3.3	-8.8 (-13.7 to -3.9)

^a^mean ± SD.

^b^44.2 ml·kg^-1 ^·min^-1 ^in men and 35.1 in women based on the results from Aspenes et al. [10].

HDL, high-density lipoprotein; LDL, low-density lipoprotein; BMI, body mass index.

### Quality of life

Results from the SF-36 questionnaire and correlations between SF-36 variables and VO_2peak _are presented in Table 3.

**Table 3:** SF-36 items scores^a ^and Pearson correlation coefficient between SF-36 items and peak oxygen uptake^b^

	Women (N = 11)		Men (N = 19)		All (N = 30)	
**SF-36 items**	**Mean ± SD**	***r***	**Mean ± SD**	***r***	**Mean ± SD**	***r***

Physical function (PF)	76.4 ± 28.1	0.68*	82.6 ± 20.2	0.48*	80.3 ± 23.1	0.58***
Role physical (RP)	56.8 ± 35.5	0.61*	67.1 ± 30.1	0.10	63.3 ± 32.0	0.34
Bodily pain (BP)	77.5 ± 24.7	0.06	73.8 ± 26.1	0.11	75.2 ± 25.2	0.26
General health (GH)	58.1 ± 17.6	0.72*	63.6 ± 19.9	0.42	61.6 ± 19.0	0.53**
Vitality (VT)	52.7 ± 22.8	0.71*	51.8 ± 18.6	0.30	52.2 ± 19.9	0.47*
Social function (SF)	51.1 ± 29.8^†^	0.41	74.3 ± 17.4	0.34	65.8 ± 25.9	0.41*
Role emotional (RE)	42.4 ± 33.7^†^	0.39	73.7 ± 37.8	0.25	62.2 ± 38.9	0.35
Mental health (MH)	55.3 ± 15.1	0.68*	67.0 ± 16.5	0.08	62.7 ± 16.7	0.34
Physical component (PCS)	49.1 ± 10.3	0.72*	48.2 ± 7.6	0.37	48.6 ± 8.6	0.51**
Mental component (MCS)	36.5 ± 8.4^†^	0.52	45.6 ± 10.2	0.16	42.2 ± 10.4	0.34

^a^Patients with schizophrenia

^b^ml·kg^-0.75^·min^-1^.

* p < 0.05, **p < 0.005, *** p < 0.001 Pearsons r. ^† ^p < 0.05 compared to men, independent T-test.

## Discussion

### Peak oxygen uptake

The present results highlight reduced VO_2peak _as a major risk factor for CVD in patients suffering from schizophrenia. The VO_2peak _was 37.1 ± 9.2 and 35.6 ± 10.7 ml·kg^-1^·min^-1 ^in men and women, respectively. These values are considerable higher than previous assumptions [3,13]. Strassnig et al. [3] reported VO_2 _values of 18.7 ± 6.8 and 13.4 ± 4.6 ml·kg^-1^·min^-1^in the men and women, respectively (mean age of 45.1 ± 10.1 years). These low VO_2peak _values is to some degree explained by the high body weight (mean BMI of 36.7 ± 7.5 m·kg^2^). However, there are some indications of an underrating of these patients' VO_2peak_. First, the patients only reached a low peak heart rate (142 ± 21 beats·min^-1^). Secondly, both Carlsson et al. [13] and Strassnig et al. [3] applied a cycle ergometer test which is known to depend more on the patients motivation than a treadmill test. Patients with schizophrenia terminate cycle tests already at submaximal work loads, in contrast to health subjects [23]. Thirdly, subjects tested on a cycle ergometer achieve 7-16% lower VO_2max _compared with a maximal treadmill test, even when HR_peak _is not significantly different [24,25].

In contrast to Strassnig et al. [3], the present results demonstrate that the mean VO_2peak _in the women was similar to the men with schizophrenia, even though the age was similar (36 years in women versus 34 years in men). Women normally have about 10 ml·kg^-1^·min^-1 ^lower VO_2peak _compared to men at the same age [10]. The mean body weight was 97.2 and 74.5 kg in men and women, respectively, which partially explain the difference in VO_2peak_.

### Comparison with healthy individuals

The comparison with normalised VO_2peak _from the HUNT Fitness study, confirm our hypothesis that VO_2peak _is reduced in men with schizophrenia. The VO_2peak _in the women with schizophrenia was almost identical (101%) to inactive healthy HUNT women. Even lower VO_2peak _in men with schizophrenia compared to normative inactive men might suggest that more than just inactivity contribute the reduced VO_2peak_. The VO_2peak _in the men with schizophrenia is similar to normative healthy men aged 60-69 years [10]. In other words, the VO_2peak _in the men with schizophrenia is comparable to healthy men that are about 30 years older. Patients with schizophrenia actually have 15-25 years shorter life expectancy than the general population [26,27]. It is noteworthy that the VO_2peak _presented in the HUNT Fitness study is somewhat higher than previous described populations with regard to objectively measured VO_2peak _[28-31].

### Cardiovascular risk

People with reduced VO_2peak _are consistently being associated with increased risk of cardiovascular and all-cause mortality. Kodama et al. [5] found that 3.5 ml·kg^-1^·min^-1 ^(1 MET) increases were associated with 13% and 15% reductions in all-cause mortality and CVD/coronary heart disease, respectively. Aspenes et al. [10] found that 5 ml·kg^-1^·min^-1 ^lower VO_2peak _correspond to 56% higher odds of having a cluster of cardiovascular risk factors.

The comparison of patients with schizophrenia below and above the VO_2peak _thresholds suggested by Aspenes et al. [10] confirm that patients below these thresholds have higher prevalence of risk factors compared with patients above the thresholds. Based on the odds ratio patients were 28.3 times more likely to have one or more risk factors if they were below the VO_2peak _thresholds. When comparing mean levels above and below thresholds, all risk factors, except glucose, was better in the patients above the thresholds. These findings suggest a strong connection between the patients VO_2peak _and the conventional risk factors for CVD, as confirmed in other populations [10,32].

Our data are not quite consistent with findings from US suggesting that especially women with schizophrenia are at high risk of developing metabolic syndrome [33]. This is most likely caused by the women's fitness level in the present study, as VO_2peak _have been described as a strong independent predictor of metabolic syndrome [32].

These results emphasize that evaluation of VO_2peak _should be incorporated into routine clinical practice for risk prediction. The prognostic value of VO_2peak _is beyond that predicted from other conventional risk factors [6,34]. Even in individuals with present risk factors, the higher levels of VO_2peak _seem to confer a significant protective effect [4]. Reduced VO_2peak _is a modifiable risk factor, and eight weeks aerobic high intensity interval training has provided significant improvements of VO_2peak _both in healthy populations [16] and in patients with schizophrenia [14]. Furthermore, to reduce the risk of CVD, the interventions are probably more dependent on improving VO_2peak _than increasing physical activity level alone [35,36].

### Quality of life

Our findings of lower SF-36 social function, role emotion and mental component score among women than among men might reflect a sex difference in the general population. Lower scores for women than for men have been identified in normative adults [37]. The gender-specific correlations between items of SF-36 and VO_2peak _suggest major gender differences in self-perception. Only the correlation with between SF-36 physical functioning and VO_2peak _was significant in men, whereas six correlations with the SF-36 were significant in women. In all subjects together the VO_2peak _correlated with the patient's perception of physical function, general health, vitality, social function, and physical component score. With some exceptions, these findings are consistent with correlations between SF-36 variables and BMI in patients with schizophrenia [38]. In line with Strassnig et al. [38] we found a significant correlation with the physical component score but not the mental component score, suggesting that reduced VO_2peak _mainly is perceived as a physical health problem, not mental. Contrary, both the mental and physical health components of QOL are found related to estimated VO_2peak _in healthy men [12]. An interesting note is, however, that the patients with lower VO_2peak _seemed to experience lower vitality and social functioning. Sedentary people are associated with greater risk of low vitality [39]. QOL are found to improve in a dose dependent manner in sedentary women when increasing physical activity level [40].

### Limitations

There are some limitations of the study. First, the sample size is low. Secondly, the patients were included in the study based on request to take part in exercise intervention studies. However, all eligible patients at the department were asked to participate in these studies. Thirdly, severe ill patients with schizophrenia, with poor insight to their illness, might have difficulties to evaluate their perception of QOL.

## Conclusions

Men with schizophrenia have lower VO_2peak _than men in the general population. Patients with a VO_2peak _below 44.2 ml·kg^-1^·min^-1 ^(men) and 35.1 ml·kg^-1^·min^-1 ^(women) have higher odds of having one or more risk factors for cardiovascular disease. Low VO_2peak _compromise patients' perceived physical health. VO_2peak _should be regarded as least as important as the conventional risk factors for CVD and evaluation of VO_2peak _should be incorporated in clinical practice. Finally, these finding represent an urging need for developing effective physical training interventions for patients with schizophrenia.

## Competing interests

The authors declare that they have no competing interests.

## Authors' contributions

GM, JH, JH and JH designed the study. JH and GEN recruited patients, performed VO_2peak _testing and other data acquisition. GM and JH undertook the statistical analysis and JH wrote the first draft of the paper. All authors have contributed to and have approved the final manuscript.

## Pre-publication history

The pre-publication history for this paper can be accessed here:

http://www.biomedcentral.com/1471-244X/11/188/prepub
